# Application of a novel peaked transparent cap to assist in rectal endoscopic submucosal dissection

**DOI:** 10.1055/a-2829-5138

**Published:** 2026-05-21

**Authors:** Junhao Liu, Xiaohong Wang, Chunhai Fu, Zhongqiong Wang, Xiaowei Tang

**Affiliations:** 1 Department of Gastroenterology 556508 The Affiliated Hospital of Southwest Medical UniversityLuzhouChina; 2 Department of Gastroenterology 159434 Xuzhou Central Hospital, Xuzhou Clinical School of Xuzhou Medical UniversityXuzhouChina; 3 Department of Endoscopic Medicine 556508 The Affiliated Hospital of Southwest Medical UniversityLuzhouChina

**Fig. 1:**
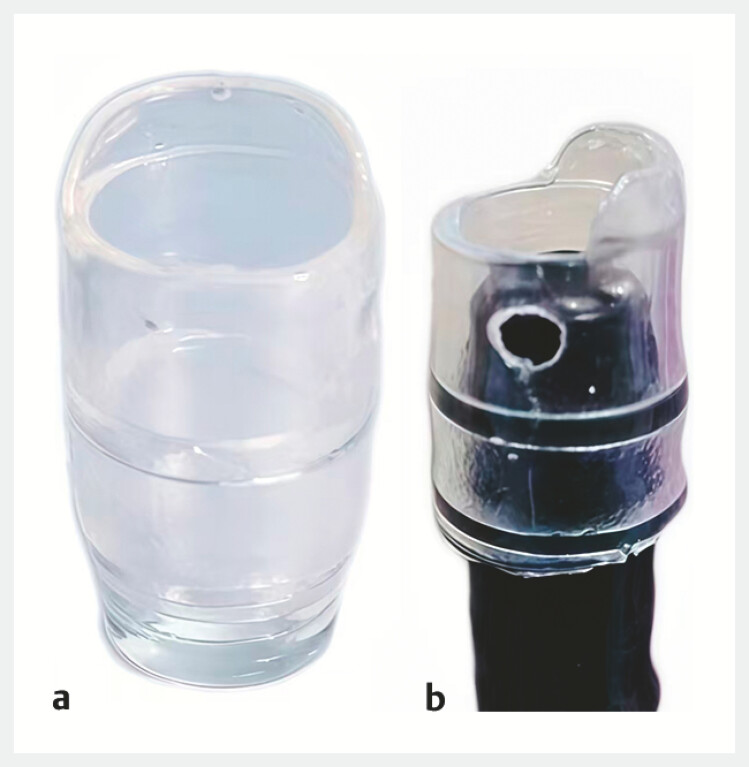
**a** and **b** The peaked transparent cap: a novel distal attachment featuring an asymmetric, peaked cap-like extension designed to provide continuous counter-traction, stabilize the mucosal flap, and maintain a clear submucosal working space during dissection; **a** the cap alone and **b** the cap mounted on the tip of the endoscope.

For endoscopic submucosal dissection (ESD), especially for large or fibrotic lesions, effective countertraction and clear visualization are essential 1 . To overcome these technical challenges, modified caps have been developed, including the calibrated small-caliber-tip hood for fibrotic lesions under saline immersion and the semi-circumferential visor cap for bubble-free underwater traction 2 3 . Here, we present a design of a peaked transparent cap (Zhejiang Shouding Medical Technology Co., Ltd) featuring a peaked cap-like extension, molded from medical-grade polyurethane using a 3D-printed (UnionTech UNIMATE) master model ( Fig. 1 ).

**Fig. 2:**
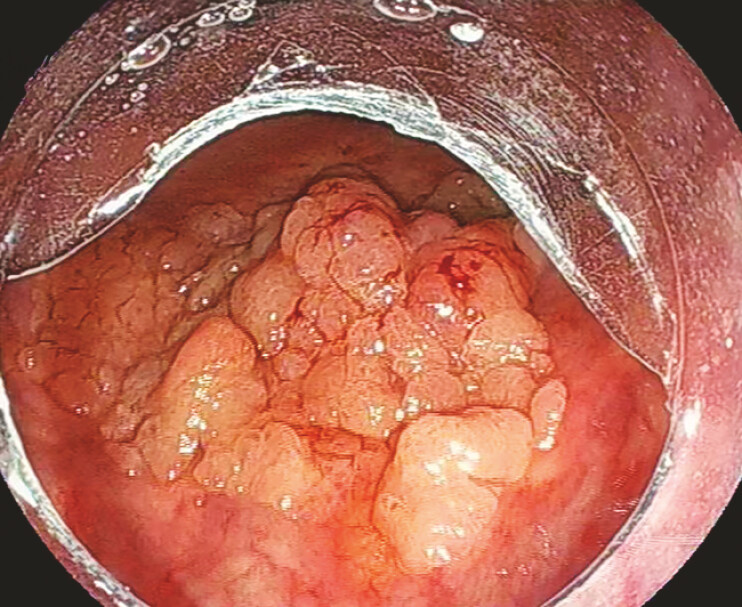
An endoscopic view of an approximately 5.0 × 3.0 cm laterally spreading tumor in the rectum, located about 5 cm from the anal verge.

**Fig. 3:**
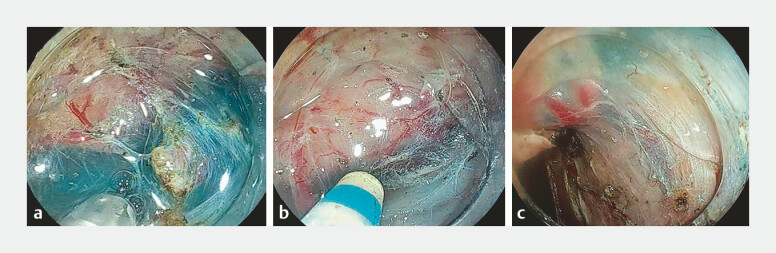
An endoscopic view during endoscopic submucosal dissection using the peaked transparent cap. **a** After submucosal injection, the mucosal, submucosal, and muscular layers are clearly delineated. **b** During dissection, the peaked cap provided continuous mucosal flap retraction, markedly improving visualization of the submucosal plane and maintaining a stable working space. **c** In the final phase of resection, the cap continued to support the dissection plane and widened the endoscopic field of view, facilitating controlled completion of en bloc resection.

**Fig. 4:**
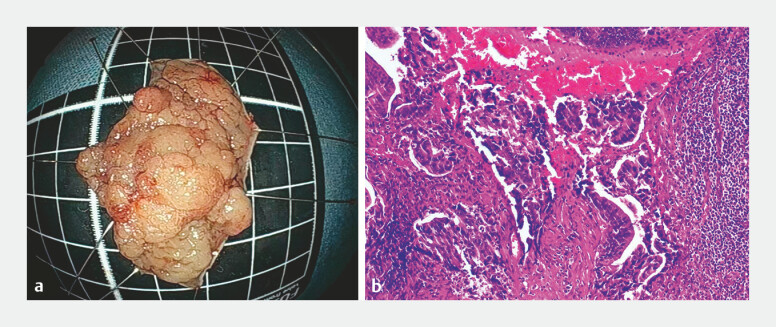
**a** The en bloc resection specimen following endoscopic submucosal dissection. **b** Histopathology revealed a villous-tubular adenoma with high-grade intraepithelial neoplasia and focal intramucosal carcinoma.

A 62-year-old woman was found to have an approximately 5.0 × 3.0 cm laterally spreading tumor (LST) in the rectum, located about 5 cm from the anal verge ( Fig. 2 ). To resect this lesion, ESD with the aid of this custom cap was performed ( Video 1 ). After submucosal injection of a methylene blue-tinted sodium hyaluronate solution for mucosal lifting, a DualKnife (ERBE, Endocut Q, Effect 3) was used to perform the mucosal incision and submucosal dissection ( Fig. 3 **a** ). The peaked cap provided continuous retraction of the mucosal flap, markedly improving visualization of the submucosal plane and maintaining a stable working space ( Fig. 3 **b** and **c** ). Using the cap as a fulcrum, the endoscope could lift the flap to optimize traction without any auxiliary devices. Hemostasis of minor bleeding was achieved with coagulation forceps (SoftCoag mode, Effect 4, 80 W). En bloc resection was accomplished without adverse events ( Fig. 4 **a** ). Histopathology revealed a villous-tubular adenoma with high-grade intraepithelial neoplasia and focal intramucosal carcinoma ( Fig. 4 **b** ).

Rectal endoscopic submucosal dissection using a novel “peaked transparent cap.”Video 1

The use of the peaked transparent cap may contribute to a more streamlined ESD procedure. By enhancing traction and improving submucosal visualization, this novel cap reduces reliance on additional devices, making the procedure more efficient and cost-effective. This innovation aids in dissection and may reduce the risk of complications, underscoring its potential to improve both the efficiency and safety of endoscopic resection for challenging lesions.

Endoscopy_UCTN_Code_TTT_1AQ_2AD_3AD
